# Supplementary material for: In situ modulating coordination fields of single-atom cobalt catalyst for enhanced oxygen reduction reaction
Source: Nat Commun. 2024 Feb 23;15:1675. doi: 10.1038/s41467-024-45990-w (PMC10891135; doi:10.1038/s41467-024-45990-w)
Supplement: Supplementary file 1 — Supplementary Information [file 41467_2024_45990_MOESM1_ESM.pdf]

Supplementary Information for

In situ modulating coordination fields of single-atom cobalt catalyst for enhanced oxygen reduction reaction

Meihuan Liu,[1,2,4] Jing Zhang,[1,4] Hui Su,[3,*] Yaling Jiang,[1] Wanlin Zhou,[1] Chenyu Yang,[1] Shuowen Bo,[1] Jun Pan,[2,*] and Qinghua Liu[1,*]

[1] National Synchrotron Radiation Laboratory, University of Science and Technology of China, Hefei 230029, Anhui, China
[2] State Key Laboratory for Powder Metallurgy, Central South University, Changsha 410083, Hunan, China
[3]Key Laboratory of Light Energy Conversion Materials of Hunan Province College, College of Chemistry and Chemical Engineering, Hunan Normal University, Changsha 410081, Hunan, China
[4] These authors contributed equally: Meihuan Liu, Jing Zhang.

*E-mail: suhui@ustc.edu.cn; jun.pan@csu.edu.cn; qhliu@ustc.edu.cn

Contents:

Supplementary Figs. 1-36;

Supplementary Tables 1-4;

Supplementary references 1-31.

Supplementary Figures and Tables.

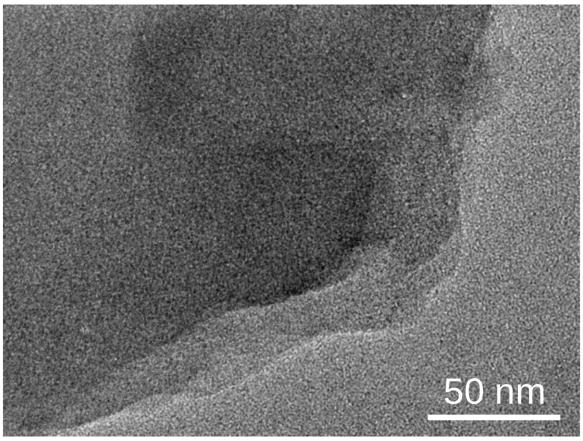

Supplementary Fig. 1. TEM image for Co/NC.

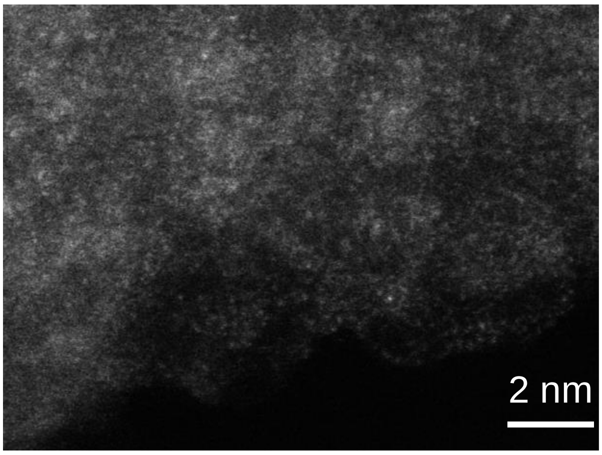

Supplementary Fig. 2. HAADF-STEM image for Co/NC.

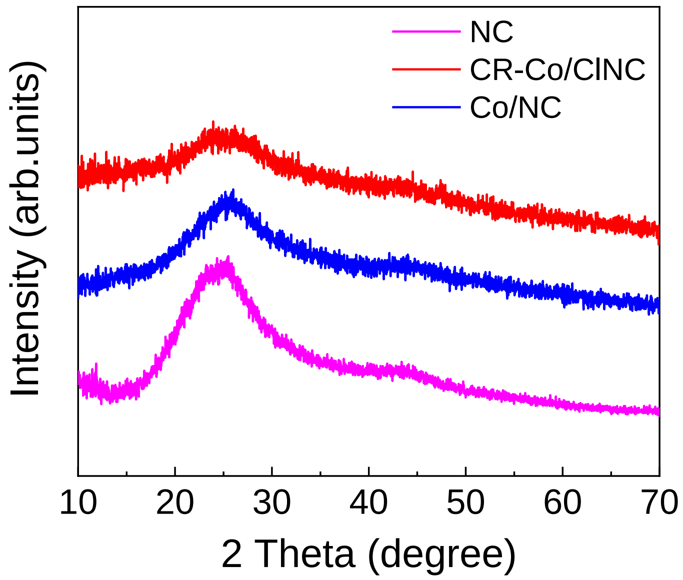

Supplementary Fig. 3. XRD patterns of CR-Co/ClNC, Co/NC, and NC.

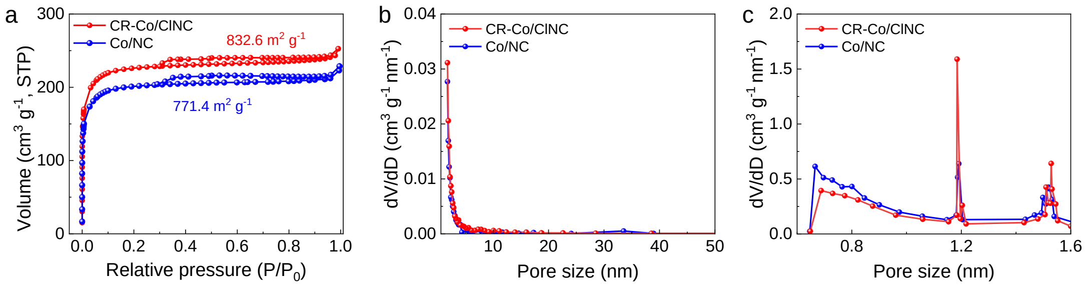

Supplementary Fig. 4. (a) Nitrogen adsorption-desorption isotherms of CR-Co/ClNC and Co/NC catalysts. Corresponding pore size distribution of CR-Co/ClNC and Co/NC catalysts based on the Barrett-Joyner-Halenda (BJH) method (b) and Horvath-Kawazoe (HK) method (c).

N_2 physisorption isotherms reveal a slightly higher specific surface area of CR-Co/ClNC and similar pore architecture in comparison with Co/NC. It shows that the accessibility of Co sites and mass transport to active sites for CR-Co/ClNC is slightly superior to Co/NC during ORR process, suggesting the significantly enhanced ORR performance of CR-Co/ClNC should therefore derive from local optimization of electronic and coordination structures.

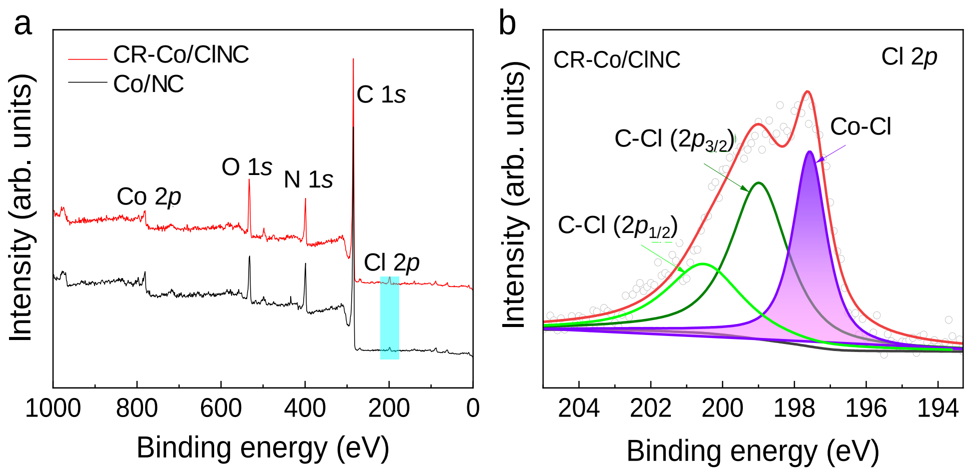

Supplementary Fig. 5. (a) XPS survey spectra of CR-Co/ClNC and Co/N and (b) Cl 2*p* XPS spectra of CR-Co/ClNC.

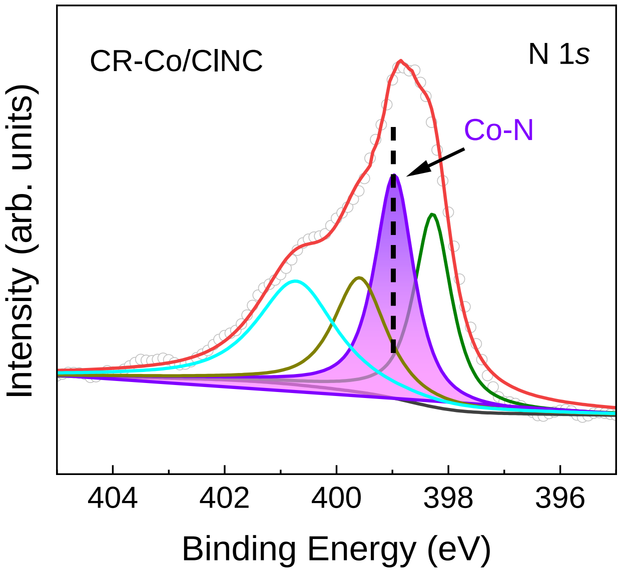

Supplementary Fig. 6. N 1*s* XPS spectra of CR-Co/ClNC.

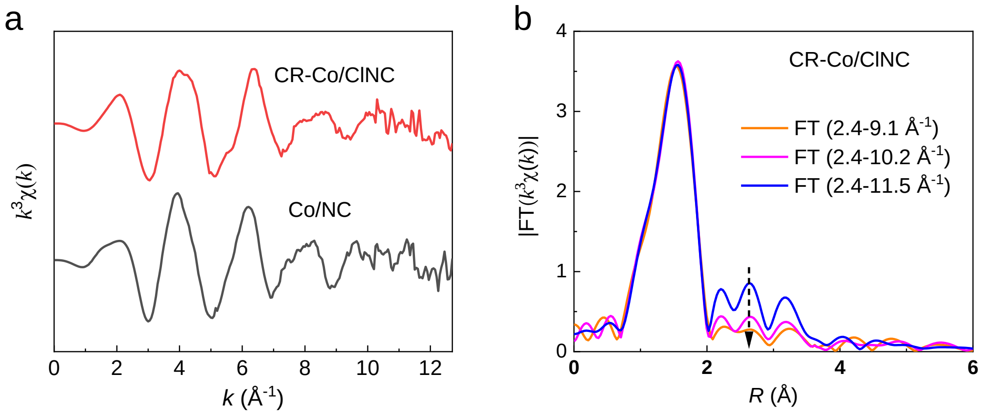

Supplementary Fig. 7. (a) $k^3\chi(k)$ curves of Co *K*-edge EXAFS oscillation functions for Co/NC and CR-Co/ClNC. (b) Corresponding k^3-weighted FT of Co *K*-edge EXAFS oscillation functions at three different *k* ranges for CR-Co/ClNC.

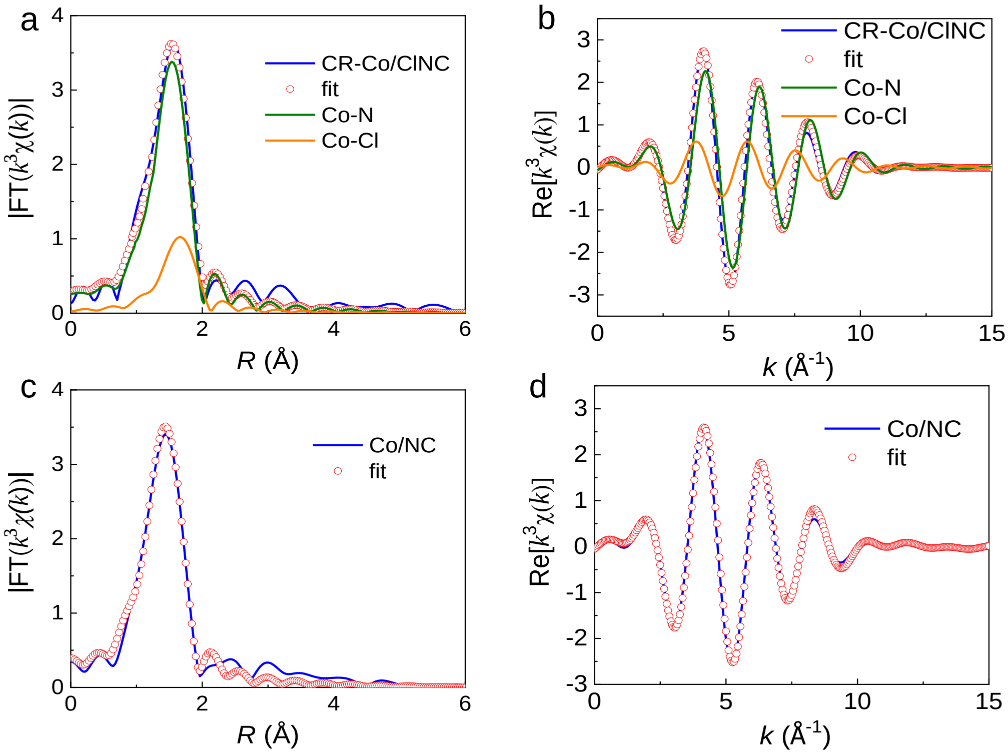

Supplementary Fig. 8. (a, c) The fitting curve of the *K*-edge k^3-weighted EXAFS spectrum and (b, d) the Re($k^3\chi(k)$) oscillation curve for CR-Co/ClNC and Co/NC, respectively.

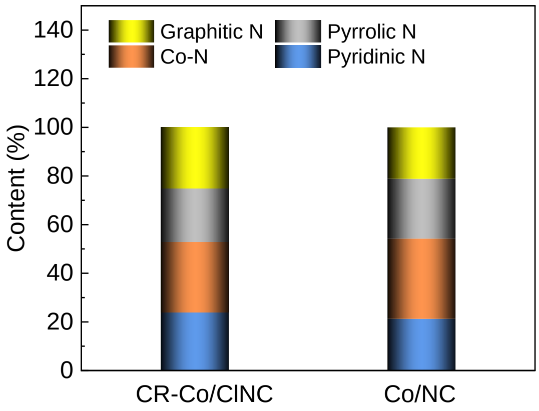

Supplementary Fig. 9. Proportions of different N species in the CR-Co/ClNC and Co/NC catalysts.

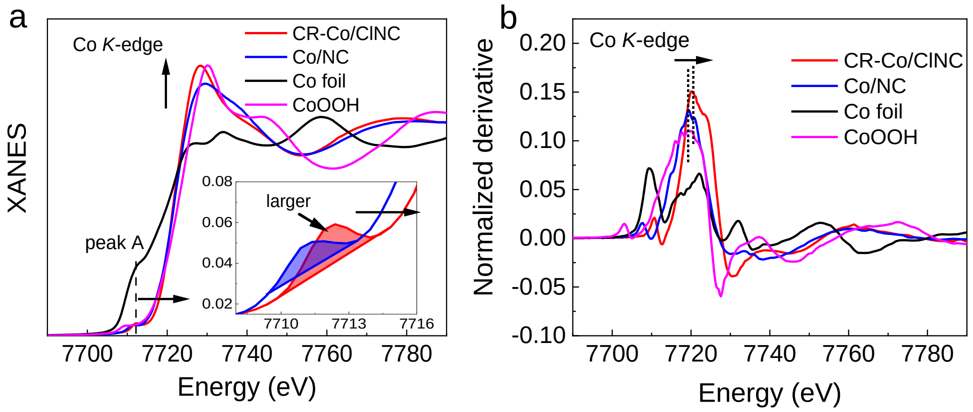

Supplementary Fig. 10. (a) Co *K*-edge XANES spectra and (b) Normalized derivative curves for CR-Co/ClNC and reference samples.

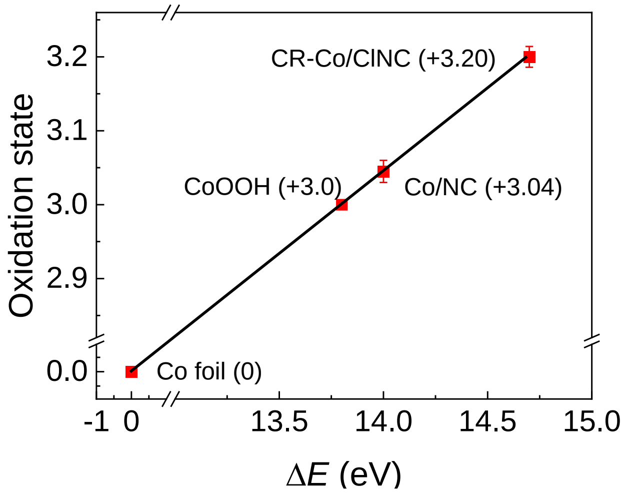

Supplementary Fig. 11. Calculated oxidation state of Co at CR-Co/ClNC and Co/NC based on the absorption edge of Co *K*-edge XANES spectra.

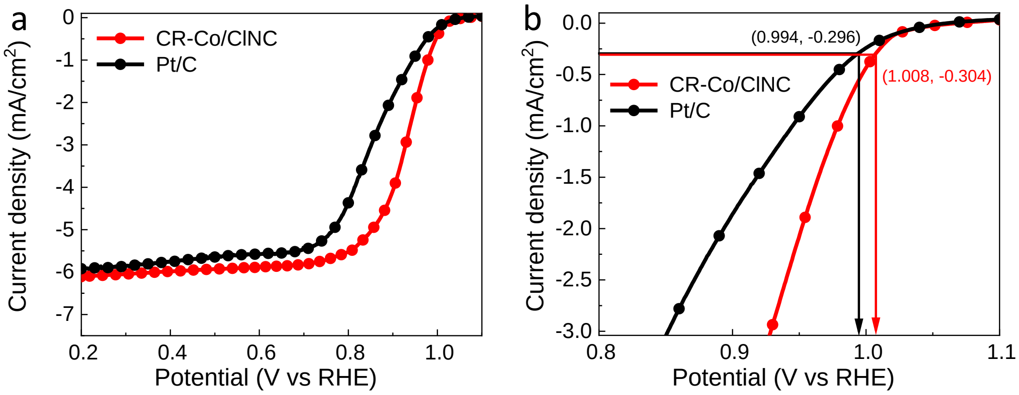

Supplementary Fig. 12. (a) Polarization curves for CR-Co/ClNC and Pt/C under 0.1 M O_2-saturated KOH, 1600 rpm. (b) The onset potential can be determined based on the LSV curves when the ORR current is 5% of the diffusion-limited current.

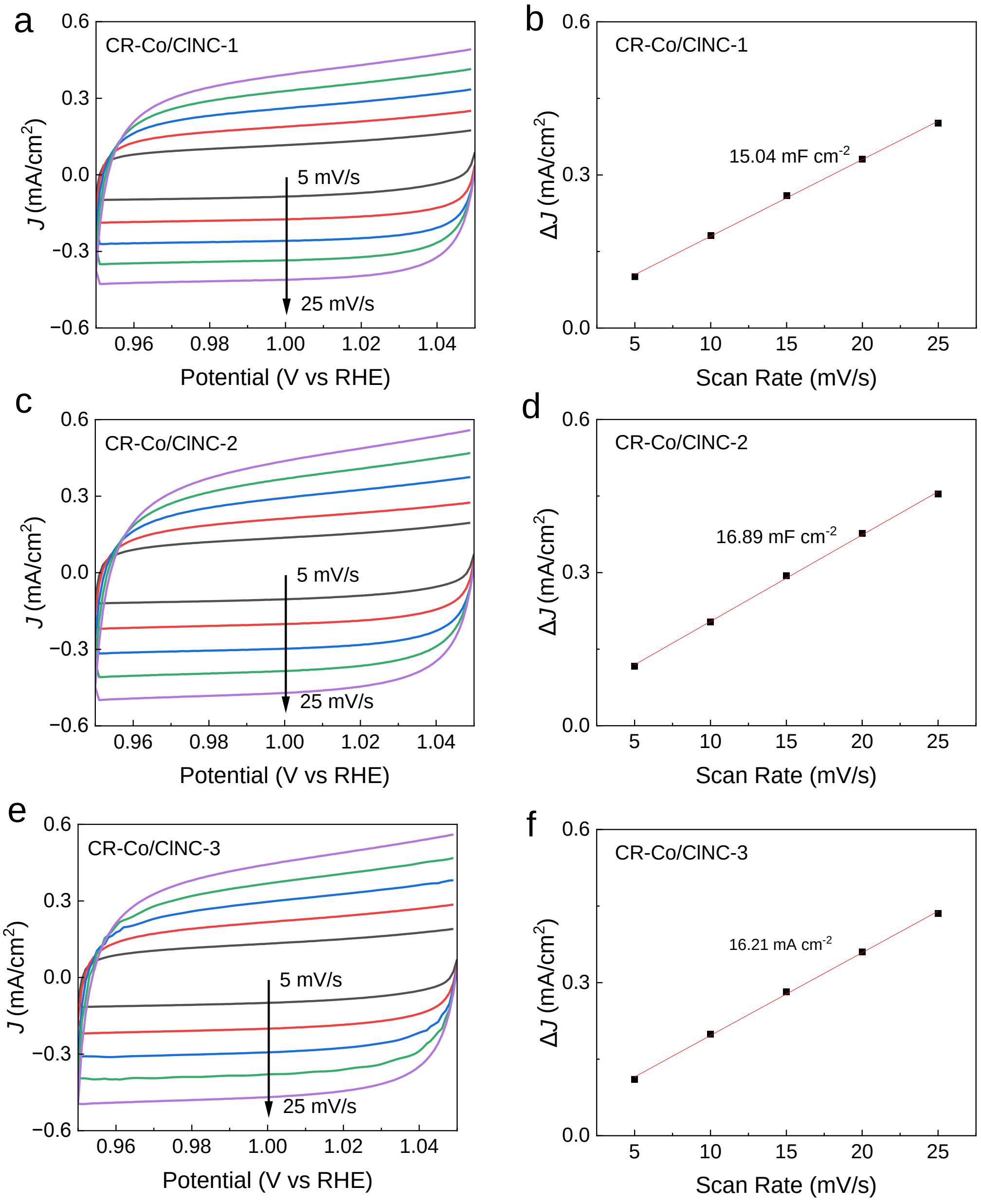

Supplementary Fig. 13. The cyclic voltammetry curves of CR-Co/ClNC catalyst at different scan rates (5–25 mV/s) (a) first, (c) second, (e) third and corresponding electrochemical double-layer capacitance (C_{dl}) (b) first, (d) second, (f) third.

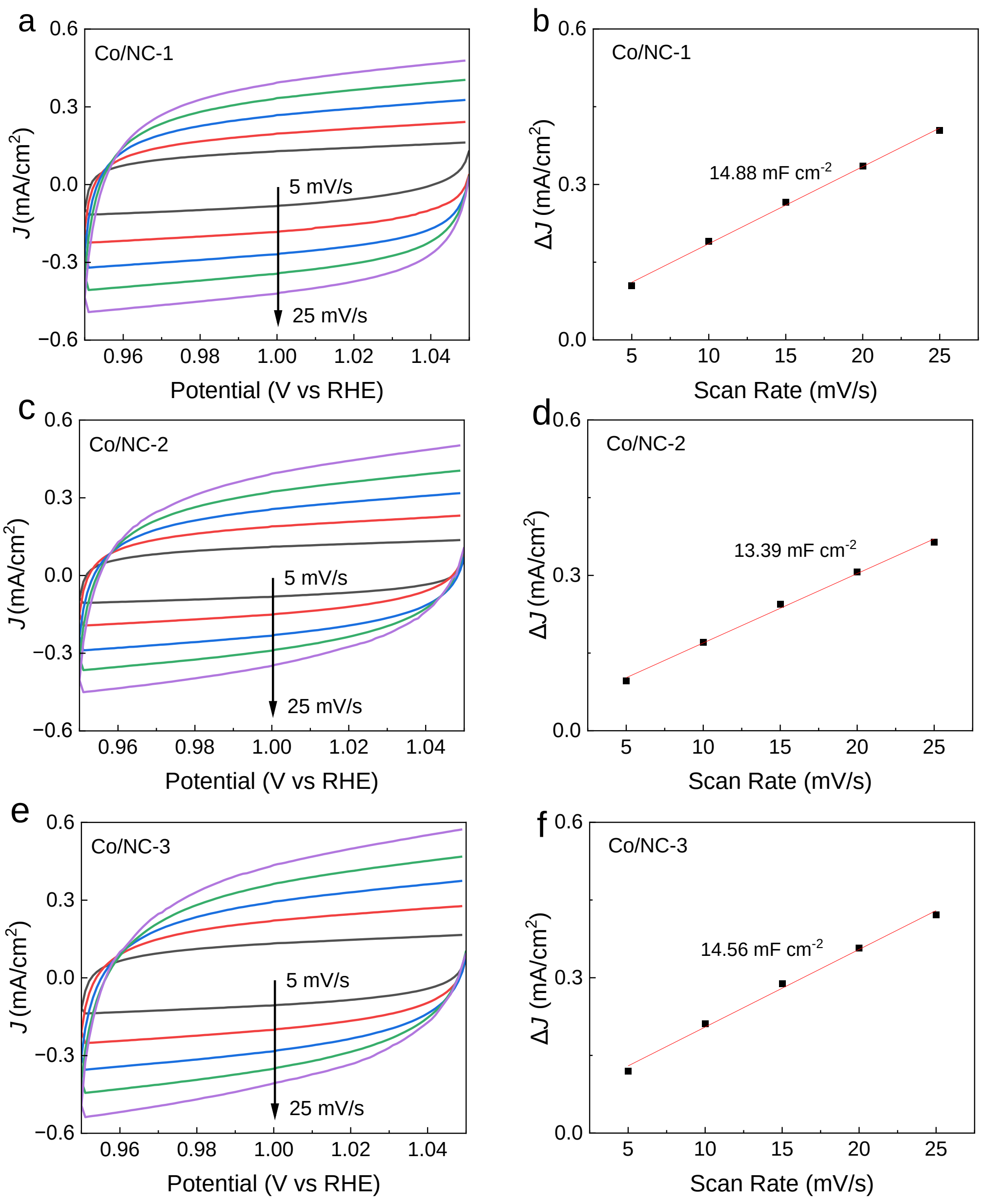

Supplementary Fig. 14. The cyclic voltammetry curves of Co/NC catalyst at different scan rates (5–25 mV/s) (a) first, (c) second, (e) third and corresponding electrochemical double-layer capacitance (C_{dl}) (b) first, (d) second, (f) third.

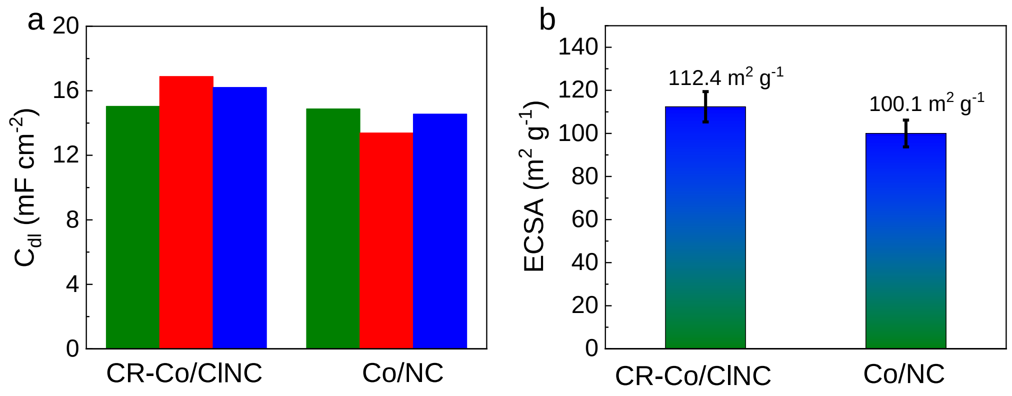

Supplementary Fig. 15. (a) C_{dl} results from three repeated tests for CR-Co/ClNC and Co/NC. (b) ECSA values calculated from C_{dl}. The error bar is obtained by three repeated tests.

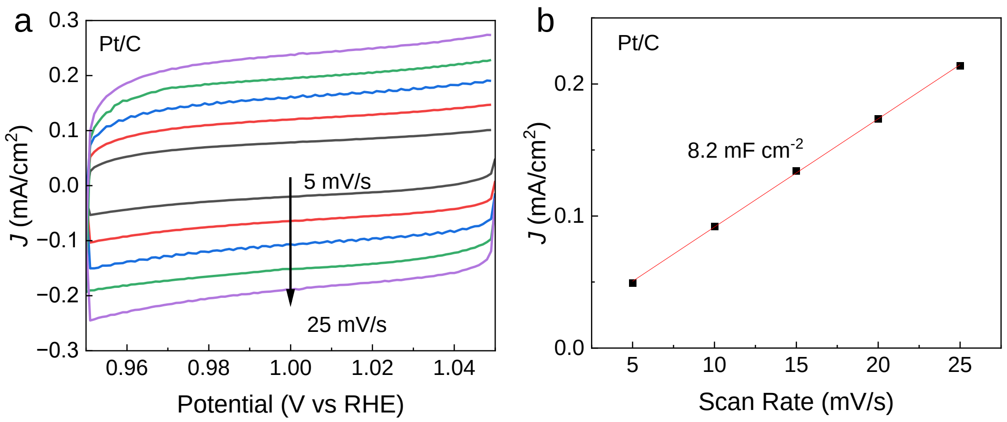

Supplementary Fig. 16. (a) The cyclic voltammetry curves of Pt/C catalyst at different scan rates (5–25 mV/s) and (b) corresponding electrochemical double-layer capacitance (C_{dl}).

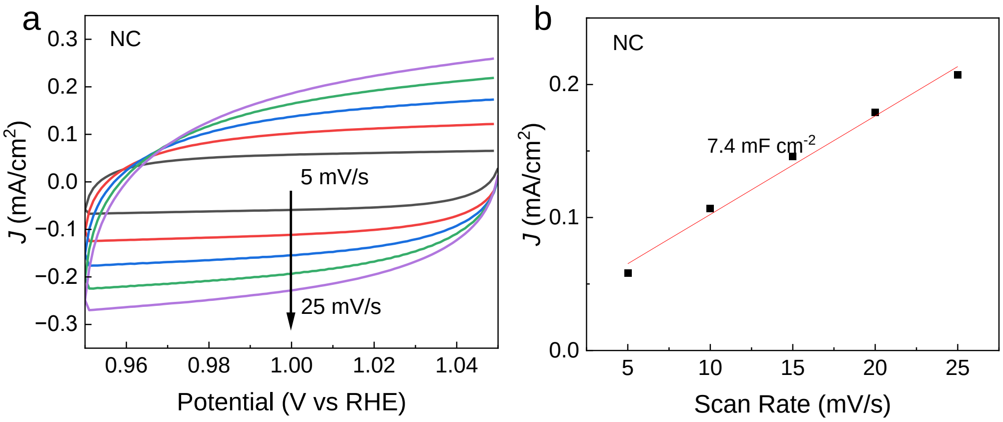

Supplementary Fig. 17. (a) The cyclic voltammetry curves of NC catalyst at different scan rates (5–25 mV/s) and (b) corresponding electrochemical double-layer capacitance (C_{dl}).

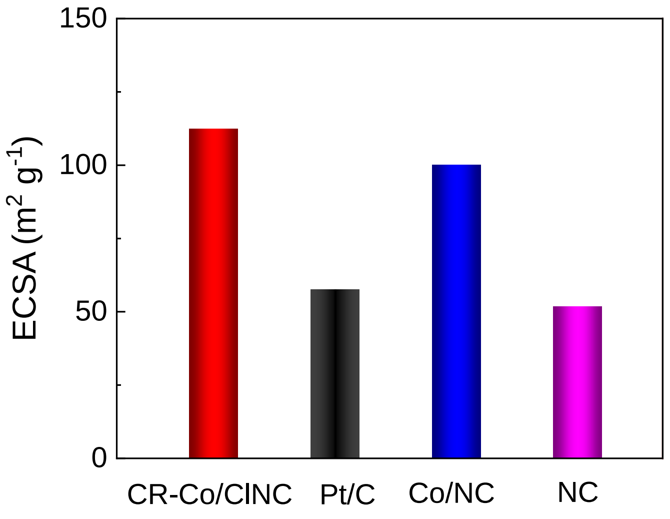

Supplementary Fig. 18. ECSA values calculated from potential cycling. The ECSA ($m^2\ g^{-1}$) can be estimated as the specific value from the gravimetric capacitance C_{dl} by the equation: $ECSA = C/(Cs \times L)$, where Cs is the double layer capacitance ($F\ m^{-2}$) of the glassy carbon electrode surface, for which the typical value of 0.4 $F\ m^{-2}$ was used in KOH solution, and L is the mass of catalyst deposited on the electrode.

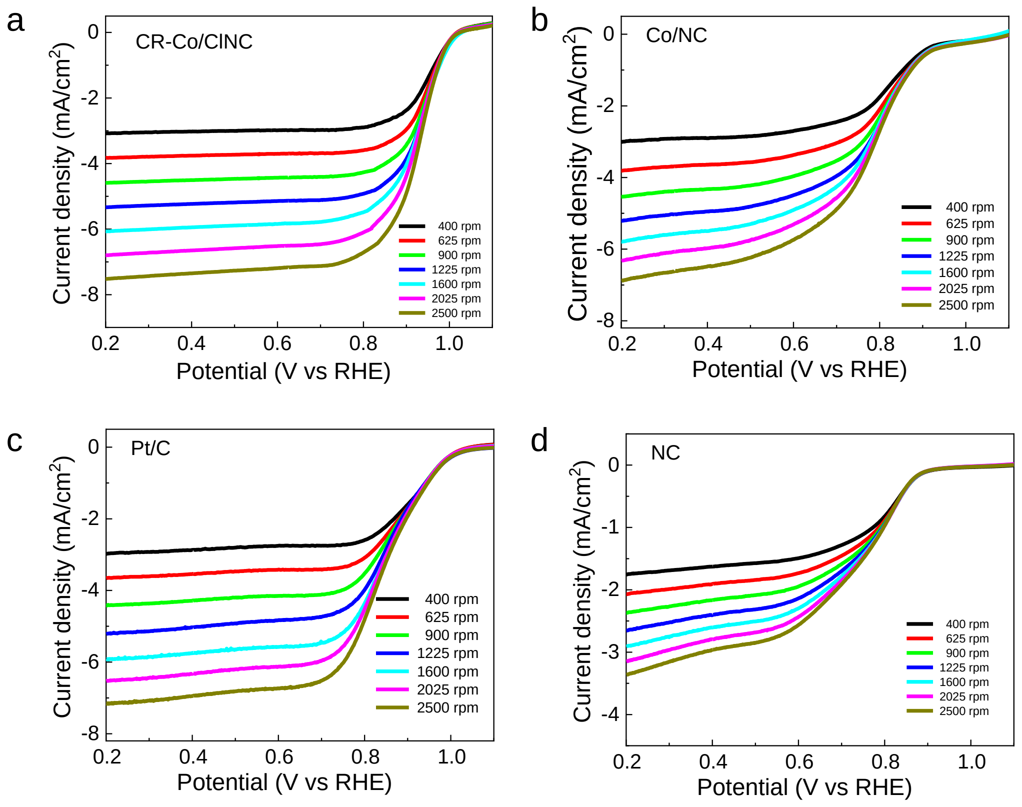

Supplementary Fig. 19. LSV curves at various rotation rates of (a) CR-Co/ClNC, (b) Co/NC, (c) Pt/C, and (d) NC.

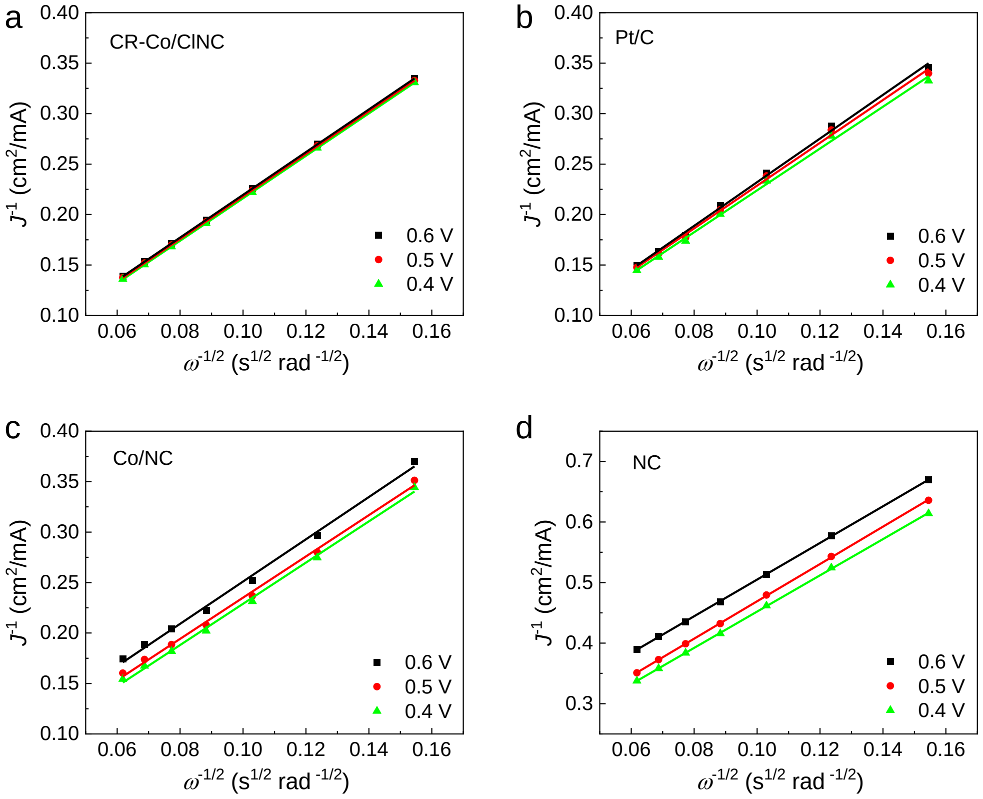

Supplementary Fig. 20. Corresponding Koutecky−Levich plots of (a) CR-Co/ClNC, (b) Co/NC, (c) Pt/C, and (d) NC.

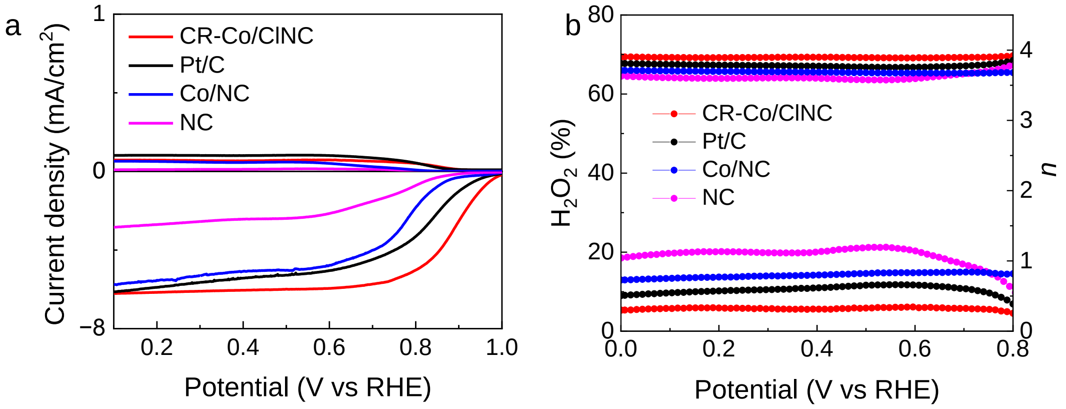

Supplementary Fig. 21. (a) RRDE curves and (b) electron number and H_2O_2 yield for CR-Co/ClNC and reference catalysts.

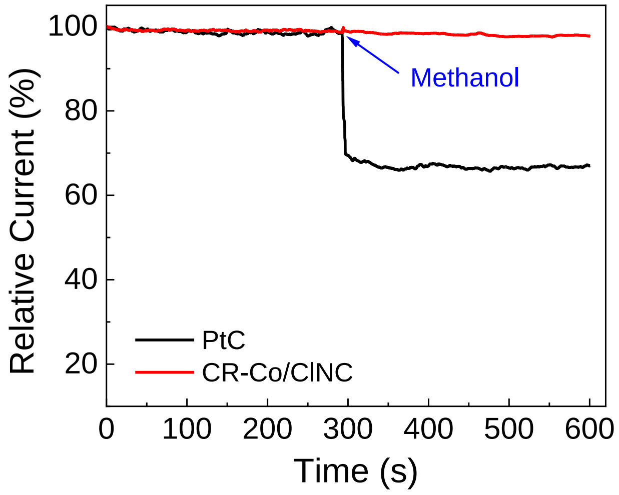

Supplementary Fig. 22. Methanol tolerance test for CR-Co/ClNC and Pt/C.

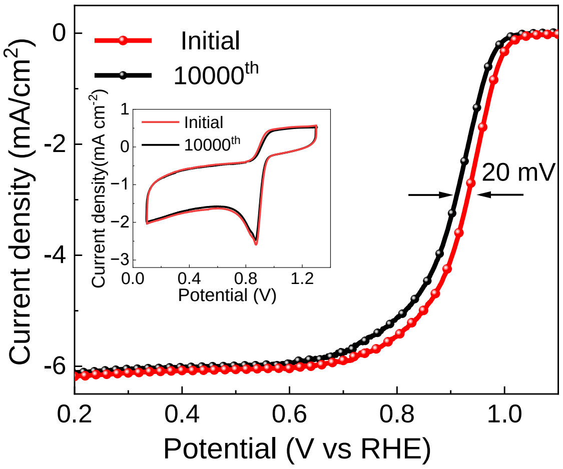

Supplementary Fig. 23. LSV curves of CR-Co/ClNC before and after durability measurement in O_2-saturated 0.1 M KOH electrolyte.

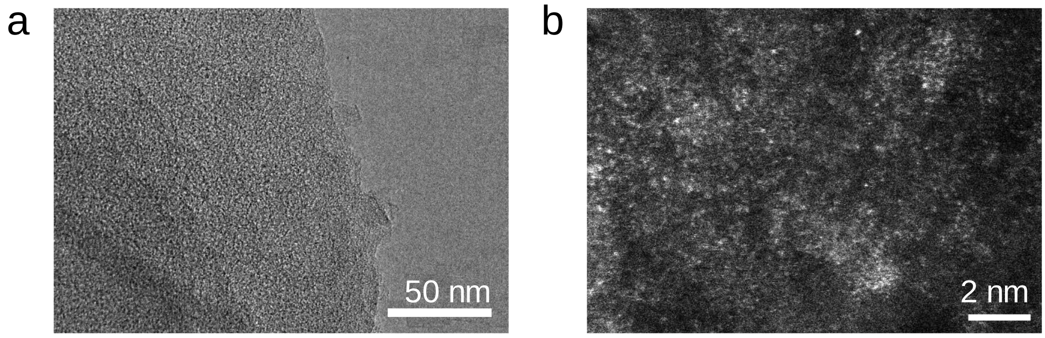

Supplementary Fig. 24. (a) TEM and (b) HAADF-STEM images of CR-Co/ClNC after durability measurement.

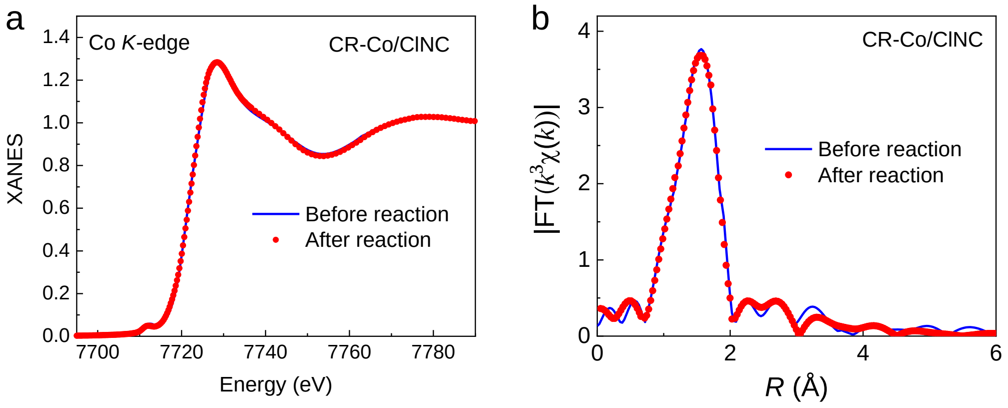

Supplementary Fig. 25. (a) Co *K*-edge XANES spectra and (b) Fourier transforms (FTs) of the Co *K*-edge EXAFS oscillations functions for the CR-Co/ClNC before and after reaction.

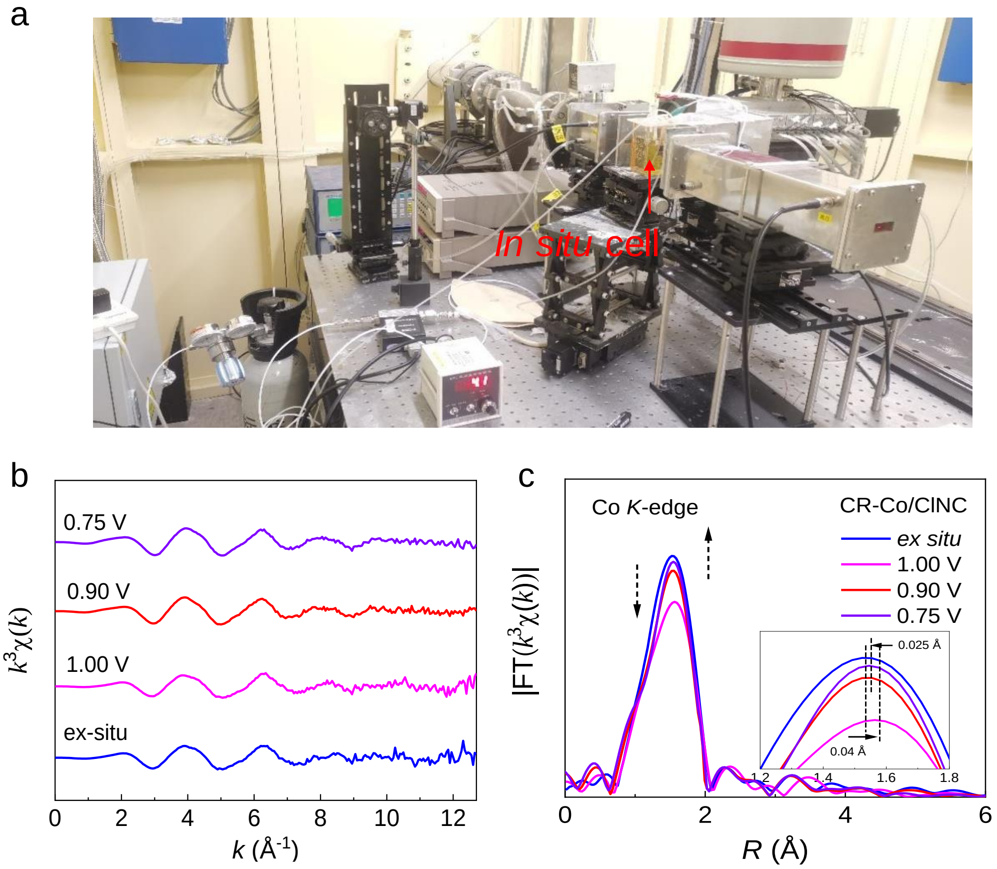

Supplementary Fig. 26. (a) *In situ* XAFS device diagram and *in situ* cell. (b) $k^3\chi(k)$ curves of Co *K*-edge EXAFS oscillation functions (c) Corresponding k^3-weighted FT of Co *K*-edge EXAFS oscillation functions for CR-Co/ClNC under different working conditions (*ex situ*, 1.00 V, 0.90 V and 0.75 V).

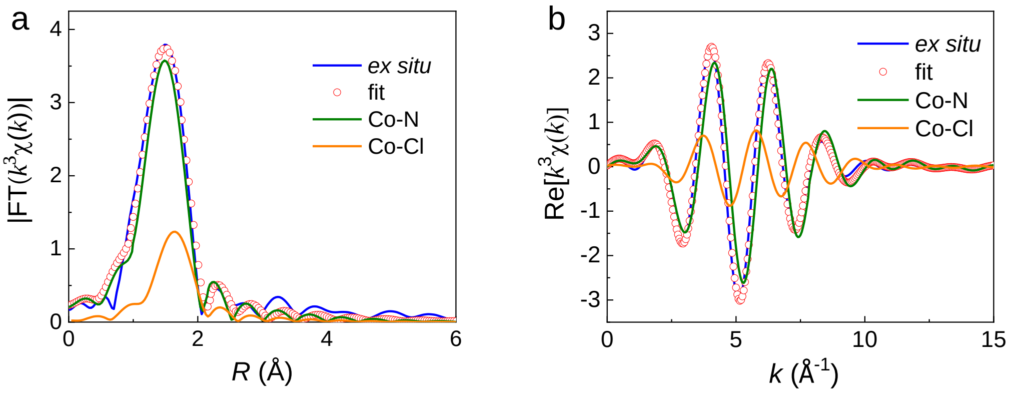

Supplementary Fig. 27. (a) The fitting curve of the Co *K*-edge k^3-weighted EXAFS spectrum and (b) the Re($k^3\chi(k)$) oscillation and fitting curve for CR-Co/ClNC under *ex situ* condition.

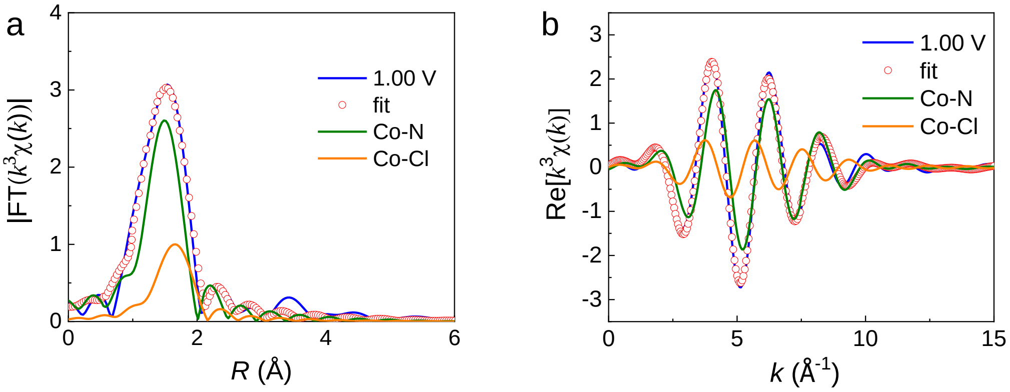

Supplementary Fig. 28. (a) The fitting curve of the Co *K*-edge k^3-weighted EXAFS spectrum and (b) the Re($k^3\chi(k)$) oscillation and fitting curve for CR-Co/ClNC under 1.00 V.

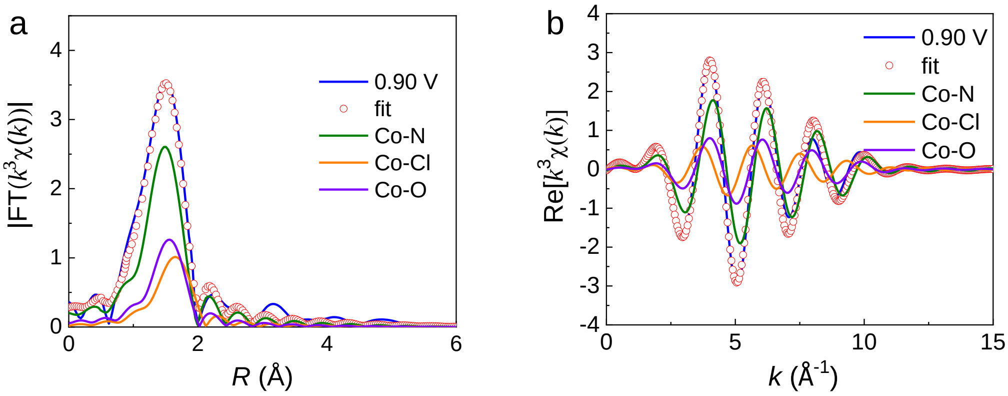

Supplementary Fig. 29. (a) The fitting curve of the Co *K*-edge k^3-weighted EXAFS spectrum and (b) the Re($k^3\chi(k)$) oscillation and fitting curve for CR-Co/ClNC under 0.90 V.

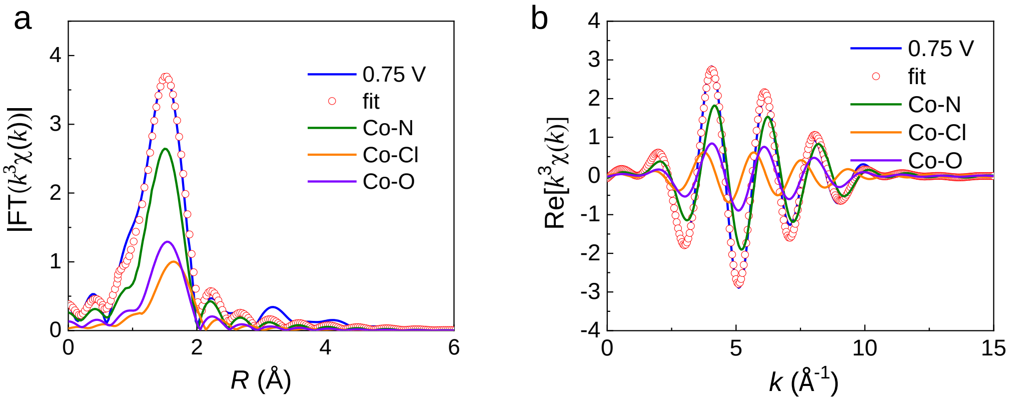

Supplementary Fig. 30. (a) The fitting curve of the Co K-edge k^3-weighted EXAFS spectrum and (b) the Re($k^3\chi(k)$) oscillation and fitting curve for CR-Co/ClNC under 0.75 V.

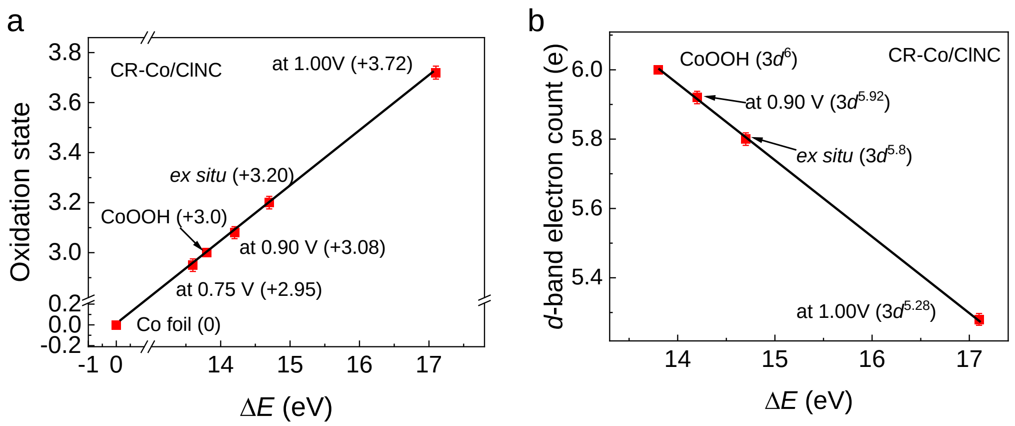

Supplementary Fig. 31. (a) The calculated oxidation state and (b) The fitted average formal *d*-band electron counts of Co at CR-Co/ClNC under *ex situ*, 1.00 V, 0.90 V and 0.70 V conditions based on the absorption edge of Co *K*-edge XANES spectra. The error bars are the standard deviations of three replicate calculation.

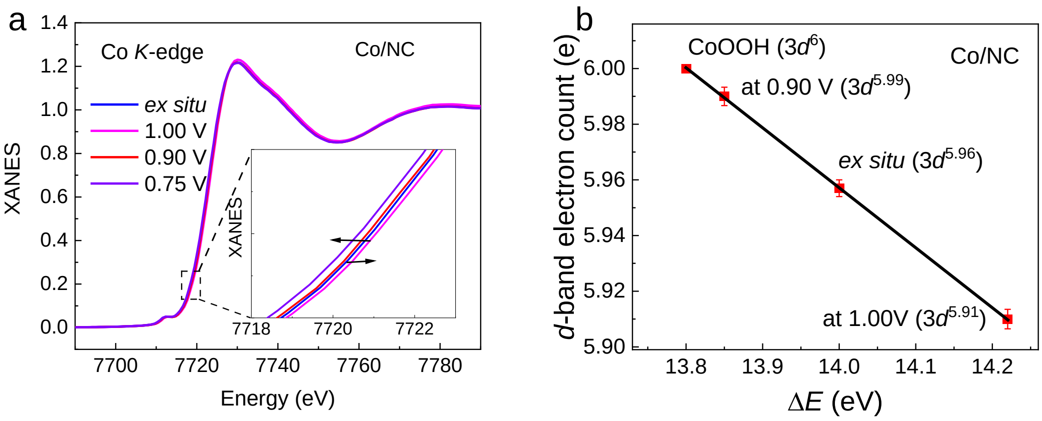

Supplementary Fig. 32. (a) XANES spectra of Co *K*-edge recorded at different applied potentials during the ORR process for Co/NC. Inset, magnified absorption edge region. (b) The fitted average formal *d*-band electron counts of Co at Co/NC under *ex situ*, 1.00 V, and 0.90 V conditions based on the absorption edge of Co *K*-edge XANES spectra. The error bars are the standard deviations of three replicate calculation.

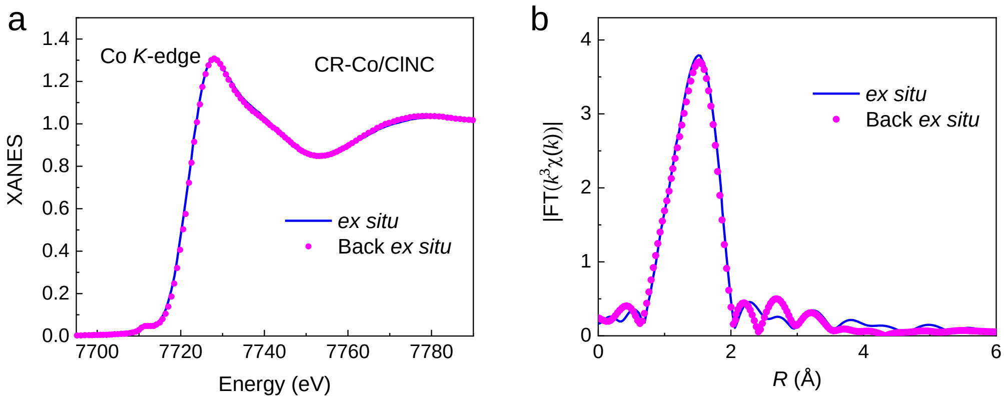

Supplementary Fig. 33. (a) Co *K*-edge XANES spectra and (b) Fourier transforms (FTs) of the Co *K*-edge EXAFS oscillations functions for the CR-Co/ClNC *ex situ* and back *ex situ* conditions.

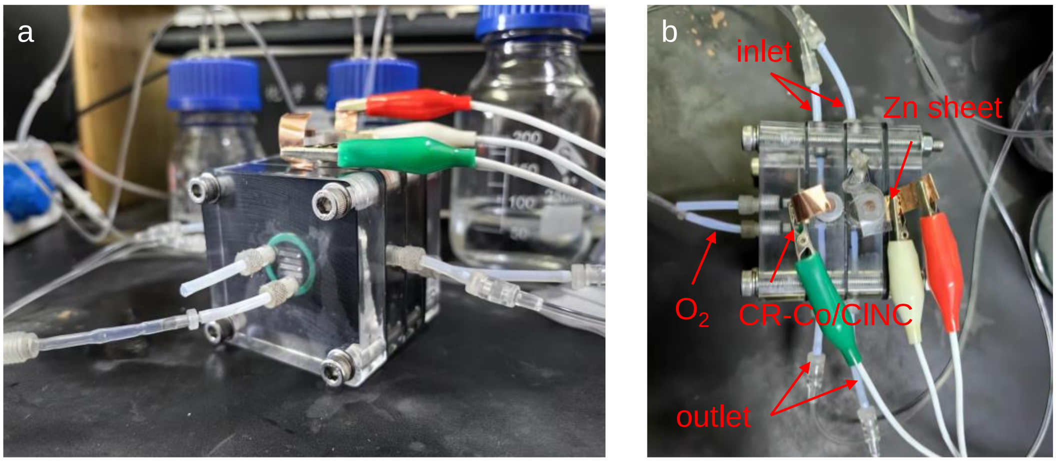

Supplementary Fig. 34. (a) Digital photograph of the Zn-air battery constructed using the CR-Co/ClNC catalyst as the cathode catalyst. (b) Top view and detail description of the homemade ZAB**.**

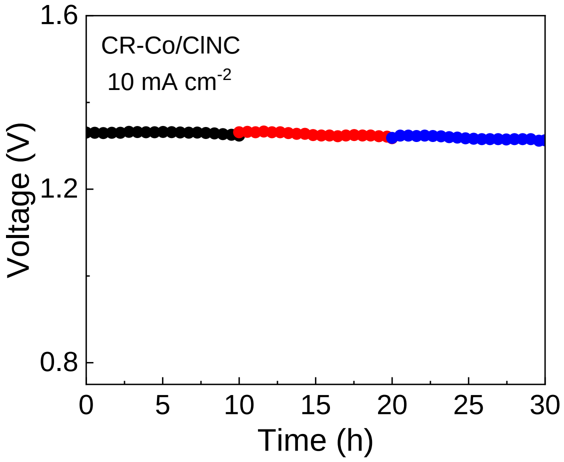

Supplementary Fig. 35. Discharging curve with three recharging cycles of CR-Co/ClNC under 10 mA cm^{-2}.

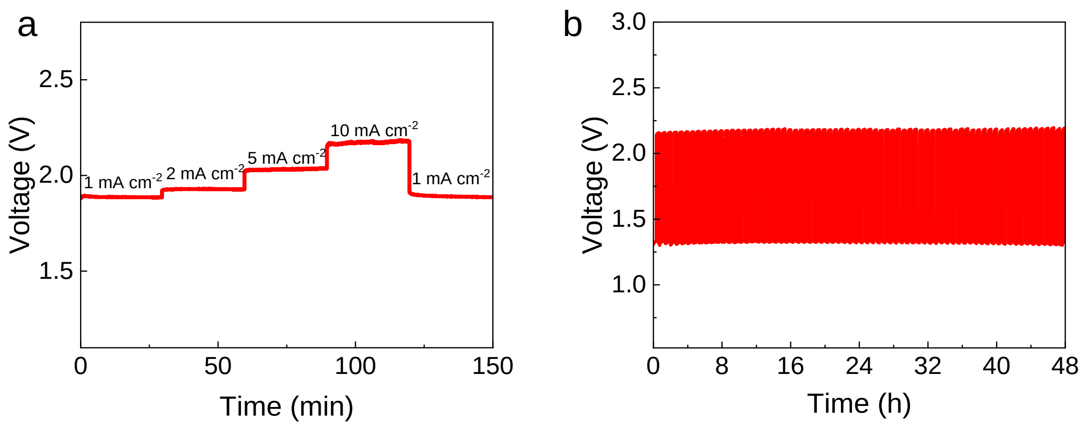

Supplementary Fig. 36. (a) Charge curves of CR-Co/ClNC based ZABs under different current densities. (b) Galvanostatic discharge-charge cycling curve performed under 10 mA cm^{-2} for the CR-Co/ClNC based ZABs.

Supplementary Table 1. Structural parameters extracted from quantitative EXAFS curve-fitting of samples using the ARTEMIS module of IFEFFIT.

Sample	Path	*N*	*R* (Å)	σ^2(10^{-3}Å^2)	ΔE_0(eV)	R-factor
CR-Co/ClNC	Co-N	3.9±0.2	2.01±0.01	8.4±0.4	-1.8±0.3	0.005
	Co-Cl	1.0±0.1	2.14±0.01			
Co/NC	Co-N	4.1±0.2	1.96±0.01	9.5±0.3	-3.0±0.6	0.005

Supplementary Table 2. Comparison of ORR performances of CR-Co/ClNC with other recently reported catalysts.

Catalysts	$E_{1/2}$ (V vs RHE)	J_k (mA cm-2)	Tafel Slope (mV dec^{-1})	References
CR-Co/ClNC	0.930	92.2@0.85 V	66	This work
Fe-N/P-C-700	0.867	24.49@0.85 V	-	1
Fe-NC SAC	0.900	-	48	2
Ce SAs/PSNC	0.900	81@0.85 V	46.8	3
Fe@Aza–PON	0.839	-	60	4
FeN_4-Te_n	0.867	16@0.85 V	86	5
Cu/Zn-NC	0.830	-	54.8	6
Se@NC-1000	0.850	-	52	7
Sb SAC	0.860	11.2@0.85 V	71.7	8
FeSA-N-C	0.900	6.1@0.90 V	-	9
Cu-SA/SNC	0.893	22.9@0.85 V	-	10

Supplementary Table 3. Structural parameters extracted from quantitative EXAFS curve-fitting of samples under working condition using the ARTEMIS module of IFEFFIT.

Sample	Path	*N*	*R* (Å)	σ^2(10^{-3}Å^2)	ΔE_0(eV)	R-factor
Ex situ	Co-N	4.0±0.1	2.01	7.9±0.8	-4.3±0.7	0.008
	Co-Cl	1.0±0.1	2.14	7.1±0.6		
1.0 V	Co-N	2.2±0.2	2.05±0.01	7.9±0.3	-5.0±0.5	0.008
	Co-Cl	1.0	2.14±0.01	6.7±0.8		
0.90 V	Co-N	2.2	2.02±0.01	7.8±0.5	-3.4±0.5	0.009
	Co-Cl	1.0	2.14	6.7±0.8		
	Co-O	1.1±0.1	2.06±0.01	7.6±0.6		
0.75 V	Co-N	2.2	2.02	7.4±0.6	-2.9±0.8	0.006
	Co-Cl	1.0	2.14	6.6±0.4		
	Co-O	1.1±0.1	2.05±0.01	7.2±0.4		

Supplementary Table 4. Comparison of Power density in ZABs of CR-Co/ClNC with other recently reported catalysts.

Catalyst	Power density (mW cm^{-2})	References
CR-Co/ClNC	176.6	This work
Co-N_4/NC	101.32	11
Fe-AC-2	153	12
Fe, Co-SA/CS	86.65	13
Fe/OES	186.8	14
Co-SAs/@NC	105.3	15
Fe-Nx-C	96.4	16
Fe-SAs/NPS-HC	195	17
Sb SAC	184.6	18
Fe/Ni-Nx/OC	148	19
FeCo-NSC	152.8	20
FeCu-SAC	201.4	21
FeNC-SAC-LS	140.18	22
FeN_5	159	23
Fe-N/P-C-700	133.2	1
Ce SAs/PSNC	212	3
Se@NC-1000	176.9	7
SACe–N/PC	155	24
CoNC-SAC	161.8	25
FeCo-DACs/NC	175	26
Fe/SNCFS-NH3	255.84	27
Fe-NiNC	220	28
o-MQFe-10: 20: 5	158.2	29
(Zn, Cu)–NC	163.8	30
Fe,Mn/N–C	160.8	31

Supplementary References

1. Yuan, K. et al. Boosting Oxygen Reduction of Single Iron Active Sites via Geometric and Electronic Engineering: Nitrogen and Phosphorus Dual Coordination. *J. Am. Chem. Soc.* **142**, 2404-2412 (2020).
2. Zhao, L. et al. Cascade anchoring strategy for general mass production of high-loading single atomic metal-nitrogen catalysts. *Nat. Commun.* **10**, 1278-1288 (2019).
3. Yin, L.et al. Heteroatom-Driven Coordination Fields Altering Single Cerium Atom Sites for Efficient Oxygen Reduction Reaction. *Adv. Mater.* **35**, 2302485 (2023).
4. Kim, S. J. et al. Defect-Free Encapsulation of Fe(0) in 2D Fused Organic Networks as a Durable Oxygen Reduction Electrocatalyst. *J. Am. Chem. Soc.* **140**, 1737-1742 (2018).
5. Ji, B. et al. Metalloid-Cluster Ligands Enabling Stable and Active FeN_4-Te_n Motifs for the Oxygen Reduction Reaction. *Adv. Mater.* **34**, 2202714 (2022).
6. Tong, M. et al. Operando Cooperated Catalytic Mechanism of Atomically Dispersed Cu-N_4 and Zn-N_4 for Promoting Oxygen Reduction Reaction. *Angew. Chem. Int. Ed.* **60**, 14005-14012 (2021).
7. Hu, H. et al. Atomically Dispersed Selenium Sites on Nitrogen-Doped Carbon for Efficient Electrocatalytic Oxygen Reduction. *Angew. Chem. Int. Ed.* **134**, e202114441 (2022).
8. Wang, T. et al. P-Block Atomically Dispersed Antimony Catalyst for Highly Efficient Oxygen Reduction Reaction. *Angew. Chem. Int. Ed.* **60**, 21237-21241 (2021).
9. Jiao, L. et al. Nanocasting SiO_2 into metal–organic frameworks imparts dual protection to high loading Fe single-atom electrocatalysts. *Nat. Commun.* **11**, 2831-2838 (2020).
10. Jiang, Z. et al. Atomic interface effect of a single atom copper catalyst for enhanced oxygen reduction reactions. *Energy Environ. Sci.* **12**, 3508-3514 (2019).
11. Chen, K. et al. Ultrasonic Plasma Engineering Toward Facile Synthesis of Single-Atom M-N_4/N-Doped Carbon (M = Fe, Co) as Superior Oxygen Electrocatalyst in Rechargeable Zinc–Air Batteries. *Nano-Micro Lett.* **13**, 60 (2021).
12. Wang, Y. et al., A gel-limiting strategy for large-scale fabrication of Fe–N–C single-atom ORR catalysts. *J. Mater. Chem. A.* **9**, 7137-7142 (2021).
13. W Jose, V. et al. Modulation of single atomic Co and Fe sites on hollow carbon nanospheres as oxygen electrodes for rechargeable Zn–air batteries. *Small Methods* **5**, 2000751 (2021).
14. Hou, C.C. et al. Single-atom iron catalysts on overhang-eave carbon cages for high-performance oxygen reduction reaction. *Angew. Chem. Int. Ed.* **132**, 7454 (2020).
15. Han, X. et al. Generation of Nanoparticle, Atomic-Cluster, and Single-Atom Cobalt Catalysts from Zeolitic Imidazole Frameworks by Spatial Isolation and Their Use in Zinc-Air Batteries. *Angew. Chem. Int. Ed.* **131**, 5413 (2019).
16. Han, J. et al. Single-atom Fe-Nx-C as an efficient electrocatalyst for zinc-air batteries. *Adv. Funct. Mater.* **29**, 1808872 (2019).
17. Chen, Y. et al., Enhanced oxygen reduction with single-atomic-site iron catalysts for a zinc-air battery and hydrogen-air fuel cell. *Nat. Commun.* **9**, 5422 (2018).
18. Wang, T. et al. P-block atomically dispersed antimony catalyst for highly efficient oxygen reduction reaction. *Angew. Chem. Int. Ed.* **60**, 21237 (2021).
19. Zhu, Z. et al. Coexisting single-atomic Fe and Ni sites on hierarchically ordered porous carbon as a highly efficient ORR electrocatalyst. *Adv. Mater.* **32**, 2004670. (2020).
20. Wu, Y. et al. Soft template-directed interlayer confinement synthesis of a Fe-Co dual single-

atom catalyst for Zn-air batteries. *Energy Storage Mater.* **45**, 805 (2022).
21. Yang, H. et al. Fe, Cu dual-metal single atom catalyst on commercial carbon black for efficient oxygen reduction reaction. *J. Mater. Chem. A.* **11**, 6191 (2023).
22. Liu, M. et al. Nanoporous-Structured Fe-NC Single-Atom Electrocatalysts Based on Lotus Seedpods and Industrial Acid Residues for Efficient Oxygen Reduction Reaction. *ACS Appl. Nano Mater.* **6**, 14831 (2023).
23. Wang, M. et al. Regulating the Coordination Geometry and Oxidation State of Single-Atom Fe Sites for Enhanced Oxygen Reduction Electrocatalysis. *Small* **19**, 2300373 (2023)
24. Li, J. C. et al. Highly dispersive cerium atoms on carbon nanowires as oxygen reduction reaction electrocatalysts for Zn-air batteries. *Nano Lett*. **21**, 4508 (2021).
25. Zhao, C.X. et al. A clicking confinement strategy to fabricate transition metal single-atom sites for bifunctional oxygen electrocatalysis. *Sci. Adv.* **8**, eabn5091 (2022).
26. Liu, M. et al. A "Pre-Constrained Metal Twins" Strategy to Prepare Efficient Dual-Metal-Atom Catalysts for Cooperative Oxygen Electrocatalysis. *Adv. Mater.* **34**, 2107421 (2022).
27. Yang, L. et al. Atomic $Fe\text{-}N_4/C$ in Flexible Carbon Fiber Membrane as Binder-Free Air Cathode for Zn-Air Batteries with Stable Cycling over 1000 h. *Adv. Mater.* **34**, 2105410 (2022).
28. Zhu, X. et al. Harnessing the interplay of Fe-Ni atom pairs embedded in nitrogen-doped carbon for bifunctional oxygen electrocatalysis. *Nano Energy* **71**,104597 (2020).
29. Liu, Y. et al. Tuning the Spin State of the Iron Center by Bridge-Bonded Fe-O-Ti Ligands for Enhanced Oxygen Reduction. *Angew. Chem. Int. Ed.* **61**, e202117617 (2022).
30. Deng, D. et al. Non-Covalent Interaction of Atomically Dispersed Cu and Zn Pair Sites for Efficient Oxygen Reduction Reaction. *Adv. Funct. Mater.* **32**,2203471 (2022).
31. Yang, G. et al. Regulating Fe-spin state by atomically dispersed Mn-N in Fe-NC catalysts with high oxygen reduction activity. *Nat. Commun*. **12**, 1734 (2021).
